# The Effects of Income Level on Susceptibility to COVID-19 and COVID-19 Morbidity/Mortality: A Nationwide Cohort Study in South Korea

**DOI:** 10.3390/jcm10204733

**Published:** 2021-10-15

**Authors:** So Young Kim, Dae Myoung Yoo, Chanyang Min, Hyo Geun Choi

**Affiliations:** 1 Department of Otorhinolaryngology-Head & Neck Surgery, CHA Bundang Medical Center, CHA University, Seongnam 13496, Korea; sossi81@hanmail.net; 2 Hallym Data Science Laboratory, Hallym University College of Medicine, Anyang 14068, Korea; ydm1285@naver.com (D.M.Y.); joicemin@naver.com (C.M.); 3 Graduate School of Public Health, Seoul National University, Seoul 08826, Korea; 4 Department of Otorhinolaryngology-Head & Neck Surgery, Hallym University College of Medicine, Anyang 14068, Korea

**Keywords:** healthcare disparities, morbidity, mortality, COVID-19, case-control studies, cohort studies

## Abstract

This study aimed to investigate the association of income level with susceptibility to coronavirus disease 2019 (COVID-19) and COVID-19 morbidity and mortality. Using the Korean National Health Insurance COVID-19 Database cohort, medical claim data from 2015 through 2020 were collected. A total of 7943 patients who were diagnosed with COVID-19 from 1 January 2020 to 4 June 2020 were included. A total of 118,914 participants had negative COVID-19 PCR tests. Income levels were classified by 20th percentiles based on 2019 Korean National Health Insurance premiums. The 20th percentile income levels were categorized into three groups (low, middle, and high). The relationship of income level with susceptibility to COVID-19 and COVID-19 morbidity and mortality was analyzed using logistic regression analysis. A high income level was related to lower odds of COVID-19 infection (adjusted odds ratio (aOR) = 0.79, 95% confidence interval (CI) = 0.75–0.83, *p* < 0.001). The negative association between income level and COVID-19 infection was maintained in all subgroups. Patients with low income levels were susceptible to COVID-19 infection; however, there was no relation of COVID-19 morbidity and mortality with income level in the Korean population.

## 1. Introduction

The coronavirus disease 2019 (COVID-19) pandemic has greatly affected many aspects of life for people around the world. Health resources were redistributed and focused on coping with severe acute respiratory syndrome coronavirus 2 (SARS-CoV-2) infection [1]. The social lockdown and restricted economic activities exposed many people to unemployment and bankruptcy. People in occupations requiring physical labor or face-to-face services, such as food service employees, supermarket or warehouse workers, telemarketers, and drivers of public transportation, selectively encountered unprecedented job loss. Conversely, individuals with contact-free jobs, such as programmers, executive officers, and capitalists, could continue their jobs from home and were less influenced by the COVID-19 epidemic. As a result, economic inequalities have been accentuated during the COVID-19 epidemic [2,3]. Increased economic inequalities are directly connected to health inequalities [4]. The disparity of income is closely related to many factors associated with socioeconomic risks, which may contribute to susceptibility to infection and COVID-19 mortality [4].

Low economic status has been highlighted as a factor affecting vulnerability to COVID-19 infection [5,6,7]. Patients with a low economic status are more likely to reside in unhealthy environments with poor hygiene. Workplaces and living spaces are unfavorable for maintaining social distancing to combat viral transmission. In addition, a diminished food industry causes shortages of food and food insecurity, which increases the risk of COVID-19 infection in people living in poverty [8]. The reproductive ratios of COVID-19 were as high as 1.29 (95% confidence intervals (CI) = 1.15–1.46) in groups with unfavorable socioeconomic status, higher than the median of 0.96 (interquartile range = 0.72–1.34) [9]. Communities with lower incomes, less insurance coverage, and more unemployment were associated with higher rates of COVID-19 in an ecological study [7]. Moreover, a low economic status may impede the early diagnosis and treatment of COVID-19, which increases the severity of disease [10]. In an ecological study, both the incidence and mortality of COVID-19 were correlated with the Gini coefficient (*rho* = +0.6906, *p* < 0.001 for the incidence of COVID-19 and *rho* = +0.6564, *p* < 0.001 for the mortality of COVID-19) [6]. To assess the relation of COVID-19 infection with economic status, other socioeconomic factors, including ethnicity, region of residence, and health insurance system, should be included in the analyses. In Korea, the diagnosis and treatment costs of COVID-19 have been completely covered by the Korean government, regardless of patients’ economic status. Thus, the Korean cohort excludes the influence of accessibility and availability of diagnosis and treatment of COVID-19 across economic levels.

We hypothesized that income level could have an impact on susceptibility to COVID-19 and on the morbidity and mortality of COVID-19. To minimize potential confounding effects, the analysis was adjusted for the covariates age, sex, and comorbidity.

## 2. Materials and Methods

### 2.1. Ethics

The Ethics Committee of Hallym University (2020-07-022) permitted this study. Written informed consent was waived by the Institutional Review Board. The participants’ information was anonymized. All analyses adhered to the guidelines and regulations of the Ethics Committee of Hallym University.

### 2.2. Study Population and Participant Selection

We used the Korean National Health Insurance COVID-19 Database (NHID-COVID DB) medical claim code data from 2015 to 2020. The NHID-COVID DB provided data for individuals who underwent SARS-CoV-2 testing, using real-time reverse transcriptase PCR assay of nasal or pharyngeal swabs, in accordance with the WHO guidelines. Control participants from the Korean National Health Insurance Database were matched by age and sex.

**Figure 1:**
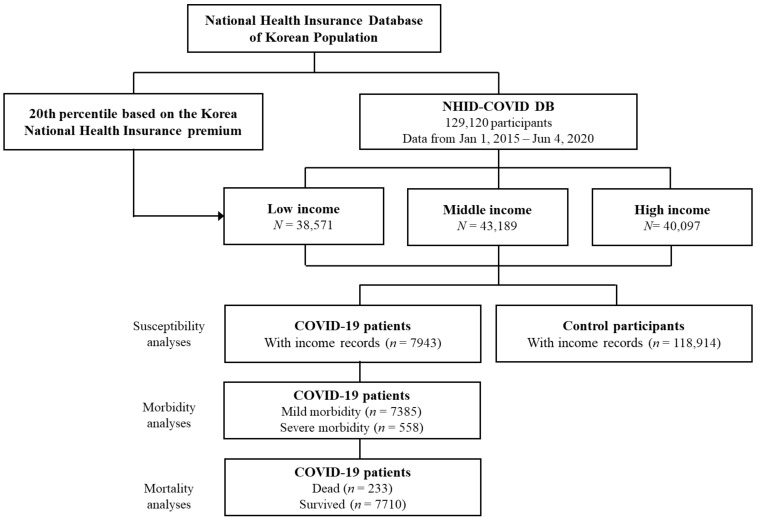
A schematic illustration of the participant selection process that was used in the present study. Of a total of 129,120 participants, 7943 COVID-19 patients and 118,914 control participants were selected.

Confirmed COVID-19 patients were included from 1 January 2020 to 4 June 2020; all of them finished treatment or died by 4 June 2020 (*n* = 8070). Fifteen times more control participants matched by age and sex were extracted (*n* = 121,050). Among them, we excluded participants with a lack of income records (*n* = 127 for COVID-19 patients, *n* = 2136 for control participants). Consequently, 7943 COVID-19 participants and 118,914 control participants were selected. Then, COVID-19 patients were analyzed for mild (*n* = 7385) and severe (*n* = 558) morbidity. They were also analyzed for death (*n* = 233) and survival (*n* = 7710) (Figure 1).

### 2.3. Exposure (Income Level)

Income level was divided into 20th percentiles based on 2019 Korea National Health Insurance premiums, ranging from 1 (the lowest 5%) to 20 (the highest 5%), for the entire Korean population with health insurance (Supplementary Table S1) [11]. In addition, medical-aid beneficiaries were added to the lowest income level, which was estimated to be approximately 3.0% of the total Korean population (class 0) [11,12]. We categorized income level into 3 groups (low (income level 0 to 6), middle (income level 7 to 14), and high (income level 15 to 20)).

### 2.4. Outcome (COVID-19 Infection)

Laboratory confirmation of SARS-CoV-2 infection, using a real-time reverse transcriptase PCR assay, was defined as the primary outcome.

### 2.5. Secondary Outcome (Morbidity and Mortality)

The secondary outcomes were morbidity and mortality in COVID-19 patients. Morbidity was defined as mild or severe. Severe morbidity was indicated by admission to the intensive care unit (ICU), invasive ventilation, extracorporeal membrane oxygenation (ECMO), or death.

### 2.6. Covariates

Age groups were divided into 10-year intervals: 0–9, 10–19, 20–29 and so on, with the oldest group being 80+ years old (total of 9 age groups).

The Charlson comorbidity index (CCI) has been widely used to measure disease burden using 17 comorbidities: myocardial infarction, congestive heart failure, peripheral vascular disease, hemiplegia or paraplegia, dementia, chronic pulmonary disease, rheumatologic disease, peptic ulcer disease, diabetes without chronic complications, diabetes with chronic complications, renal disease, any malignancy, including leukemia and lymphoma, metastatic solid tumor, mild liver disease, moderate or severe liver disease, and HIV/AIDS [13]. The presence of each comorbidity was counted with a weighted value and summed as a CCI score. It is a continuous variable (0 (no comorbidities) through 29 (multiple comorbidities)) [13]. In addition, hypertension (ICD-10 codes: I10 and I15) was assigned if participants were treated ≥2 times, as it was not included in the CCI.

### 2.7. Statistical Analyses

The general characteristics of all participants were compared among income groups using the chi-squared test.

To estimate the susceptibility to COVID-19, of COVID-19 patients compared to control participants, odds ratios (ORs) with 95% confidence intervals (CIs) of income were calculated using crude (simple model) and adjusted (for age, sex, CCI score, and hypertension) logistic regression models. To estimate morbidity/mortality in COVID-19 patients by income, logistic regression was used. For subgroup analyses, we divided participants by age (<50 years old and ≥50 years old), sex, CCI score (0 score, 1 score, and ≥2 score), and hypertension history.

For the statistical analyses, SAS version 9.4 (SAS Institute Inc., Cary, NC, USA) was used. We performed two-tailed analyses, and significance was defined as *p* values less than 0.05.

## 3. Results

The prevalence of COVID-19 was different among income groups (*p* < 0.001, Table 1). Totals of 7.4% (2836/38,571), 5.8% (2489/43,189), and 5.8% (2618/40,097) of the low-, middle-, and high-income groups had histories of COVID-19. The morbidity of COVID-19 was 6.5% (185/2836), 6.5% (161/2489), and 8.1% (212/2618) for the low-, middle-, and high-income groups, respectively (*p* = 0.03). The mortality of COVID-19 was 0.23% (86/2836), 0.14% (62/2489), and 0.19% (85/2618) for the low-, middle-, and high-income groups, respectively (*p* = 0.03). The distributions of age, sex, CCI score, and history of hypertension were different among income groups (all *p* < 0.001).

Income level was inversely related to susceptibility to COVID-19 (Table 2). Compared to the low-income group, the middle- and high-income groups demonstrated lower odds of COVID-19 infection (adjusted OR (aOR) = 0.78, 95% CI = 0.74–0.83, *p* < 0.001 for the middle-income group and aOR = 0.79, 95% CI = 0.75–0.83, *p* < 0.001 for the high-income group). According to the analysis of 20th percentile income levels, ranging from 1 (the lowest 5%) to 20 (the highest 5%), a high income level was associated with 0.98 times lower odds of COVID-19 infection (95% CI = 0.98–0.99, *p* < 0.001). Additional analyses according to age, sex, CCI score, and history of hypertension showed a consistent association of COVID-19 infection with lower income (Supplementary Table S2).

The morbidity of COVID-19 was not associated with income level in the adjusted models (Table 3). The high-income group showed 1.26 times higher odds of COVID-19 morbidity in the crude model (95% CI = 1.03–1.55, *p* = 0.03); however, there was no significant association of COVID-19 morbidity with income level when adjusted for age, sex, CCI score, and hypertension. Among the age, sex, CCI score, and history of hypertension subgroups, males with no past medical history (CCI score = 0), and an absence of hypertension, had higher odds of COVID-19 morbidity in the higher income groups (Supplement Table S3). The middle-income level demonstrated 1.49 times higher odds of COVID-19 morbidity than the low-income level in the male group (95% CI = 1.06–2.07, *p* = 0.03). The group with no past medical history and the hypertension-free group showed 1.03 times (95% CI = 1.01–1.05, *p* = 0.004) and 1.02 times (95% CI = 1.00–1.04, *p* = 0.03) higher odds of COVID-19 morbidity with higher income levels, respectively.

COVID-19 mortality was not associated with income level (Table 4). Neither the middle- nor high-income groups showed increased odds of mortality due to COVID-19 (aOR = 1.09, 95% CI = 0.75–1.58, *p* = 0.65 for the middle-income group and aOR = 0.76, 95% CI = 0.54–1.08, *p* = 0.19 for the high-income group). None of the 20th percentile income levels were related to mortality due to COVID-19 (aOR = 0.99, 95% CI = 0.97–1.01, *p* = 0.15). None of the age, sex, CCI score, or history of hypertension subgroups showed an association between COVID-19 mortality and income level, except for the group with a CCI score = 1 (Supplementary Table S4). In the CCI score = 1 group, the high-income group had 0.43 times lower odds of mortality due to COVID-19 (95% CI = 0.22–0.83, *p* = 0.01).

## 4. Discussion

### 4.1. Principal Results

A lower income level was associated with a higher susceptibility to COVID-19 infection; however, COVID-19 morbidity and mortality were not related to income level in the overall population. The mortality of COVID-19 was lower in the high-income group in the CCI score = 1 subgroup. However, the morbidity of COVID-19 was higher at high income levels in the male sex, CCI score = 0, and hypertension-free subgroups. The present results indicated an increased susceptibility to COVID-19 infection in lower-income-level participants; therefore, a correlation mostly likely exists between economic inequality and COVID-19 susceptibility. The present study improved upon previous studies by analyzing susceptibility to COVID-19 and COVID-19 morbidity and mortality in the same national cohort. This study examined the impact of economic level on susceptibility to COVID-19 and COVID-19 morbidity and mortality in the absence of disparities in the availability of medical resources.

### 4.2. Comparison with Prior Work

A number of previous studies suggested a higher susceptibility to COVID-19 infection in lower economic groups [5,6,7,9,14]. A retrospective study in a European urban area showed increased incidences of COVID-19 in low-income groups (risk ratio (RR) = 1.67, 95% CI = 1.41–1.96 for men and RR = 1.71, 95% CI = 1.44–1.99 for women) [14]. A low income level could influence susceptibility to COVID-19 via an elevated risk of viral exposure and an immune system that is impaired during the neutralization of a viral infection. An increased possibility of viral exposure could be linked to a higher risk of COVID-19 infection in the low-income population. Adverse living and working environments may increase the risk of COVID-19 infection in low-income populations. Poverty and one’s physical environment, such as a homeless status and/or exposure to smoking, are social determinants of health and have an impact on COVID-19 outcomes [15]. Crowded living conditions, poor hygiene, less access to healthcare, and quarantining can increase the risk of viral infection in homeless populations [16]. The low-income group exhibited less social distancing during the COVID-19 pandemic [17]. As much as approximately 36.0% (147/408) of the homeless population in Boston tested positive for SARS-CoV-2, using PCR testing [18]. The group with a low socioeconomic status demonstrated a strong association of COVID-19 infection with current smoking (aOR = 3.53, 95% CI = 1.22–2.62) [19].

In the present study, the income level was classified based on the health insurance premium, which reflected the income quintile. All Koreans must be registered with the national health insurance system; therefore, the classified income levels were precise. Korea was ranked as the country with the 12th highest gross domestic product (GDP) worldwide in 2017 [20]. Compared to other countries with similar GDP levels, such as Italy, Australia, and Spain, Korea showed a lower rate of contraction of SARS-CoV-2 and a lower mortality rate for COVID-19. A number of features, including a strong central autonomous agency that used research for agile and responsive policymaking, public trust in government measures, strong public–private sector collaboration, and surveillance and response built on integrated information management systems, could contribute to the lower infection rate and mortality rate of COVID-19 in Korea [21]. In addition, the Korean government covered all medical costs for COVID-19, enabling all participants to be examined and treated without discrimination. Additional factors contributing to socioeconomic deprivation could affect susceptibility to COVID-19, such as occupation, educational level, housing status, and food security, which were not available in the present cohort [22]. Another Korean epidemiological study suggested increased susceptibility to COVID-19 in participants with less healthcare access, less education, more risky health behaviors, crowding, specific comorbidities, difficulty social distancing, and population mobility [23].

Decreased immune system ability to combat SARS-CoV-2 infection could increase susceptibility to COVID-19 in low-income populations. Pre-existing health inequalities could add to the risk of COVID-19 infection in low-income populations. Low socioeconomic status was associated with a higher rate of chronic diseases, which made individuals with that status more vulnerable to COVID-19 [24]. Comorbidities, including diabetes and kidney diseases, have been associated with higher COVID-19 morbidity [25,26]. The overall comorbidity burdens were estimated to be approximately 1.3 times higher for hospitalization for COVID-19 in white patients (95% CI = 1.11–1.53, *p* = 0.001) [25]. In addition, an increased stress level may diminish immune functioning in the low-income group [5]. A weakened immune system could increase invasion by and replication of SARS-CoV-2 in this population. Low socioeconomic status was associated with perceived stress and health-risk behavior in a cross-sectional study (aOR = 2.90, 95% CI = 2.53–3.33 for perceived stress) [27], and the COVID-19 epidemic is likely to have a higher impact on the economic status of low-income groups because it may impose higher stress on these groups. In fact, during the COVID-19 pandemic, the lower-income group developed severe psychological distress more often than the higher-income group in a longitudinal study (aOR = 3.00, 95% CI = 1.01–9.58) [28]. Acute stress and chronic stress tended to suppress cellular and humoral immunity in a meta-analysis [29]. Thus, high stress in the low-income group could increase susceptibility to COVID-19.

The morbidity and mortality of COVID-19 did not show an association with income level in this study. In contrast, several retrospective studies reported a higher risk of severe illness from COVID-19 in low-income populations [30,31]. In a U.S. study, the low-income group had a higher risk of severe illness from COVID-19 than the higher-income groups (prevalence ratios = 1.63, 95% CI = 1.59–1.67) [30]. Moreover, the initial severity of COVID-19 was higher in patients residing in a poor district of Paris (aOR = 1.099, 95% CI = 1.038–1.178) [31]. Full coverage of COVID-19 treatment costs may have minimized the cases of undertreatment in our cohort. In Korea, the medical costs related to the diagnosis and treatment of COVID-19 have been covered by the Korean government. The indemnity of insurance coverage was suggested to improve the opportunity for regular healthcare compared to the uninsured population [32]. In a propensity-score-matched case-control study, uninsured adults showed higher mortality than insured adults (adjusted hazard ratio = 1.43, 95% CI = 1.10–1.85, *p* = 0.01) [33]. Thus, patient income levels are unlikely to affect the procedures involved in COVID-19 therapy. Moreover, the relatively small number of COVID-19 cases with morbidity and mortality may attenuate the statistical power to delineate the association of income level with morbidity and mortality.

### 4.3. Limitations

The present study used a nationwide, representative cohort. Our cohort comprised a single ethnicity (Korean); therefore, the possible impacts of ethnic disparities on outcomes were minimized [34]. In addition, the bias from undetected or undertreated COVID-19 cases was likely minimized in our cohort because the Korean government diagnosed and treated COVID-19 without any charge. Healthcare resources were never in short supply in Korea, and the infection rate of SARS-CoV-2 was controlled at less than 2000 persons per day. Thus, the diagnosis and treatment of COVID-19 were not influenced by individual economic status in this study. However, a few limitations should be considered when applying the present results. Although adjustments were made for age, sex, and comorbidities, confounders for COVID-19 infection remained, such as occupation and region of residence. Information on occupation and region of residence was not available in the NHID-COVID DB to guarantee the participants’ anonymity. These remaining confounders could have influenced the positive association of COVID-19 morbidity with high income levels in some subgroups in this study. This study included patients with COVID-19 from 1 January 2020 to 4 June 2020. This period was in the early COVID-19 pandemic period; therefore, the long-term effects of income level on COVID-19 infection need to be evaluated in further studies.

## 5. Conclusions

COVID-19 infection was higher in participants with lower income levels in the Korean population; however, the mortality of COVID-19 was not different according to income level in Korea. Public and government management of COVID-19 may impact the association of COVID-19 with income level. Health inequalities can be aggravated by a high rate of COVID-19 infection in deprived populations; therefore, active and prompt measures are essential.

## Figures and Tables

**Table 1:** General characteristics of the participants.

Characteristics	Total Participants			
	Low-Income Group (*n*, %)	Middle-Income Group (*n*, %)	High-Income Group (*n*, %)	*p*-Value
Total number	38,571 (100.0)	43,189 (100.0)	45,097 (100.0)	
Age (years old)				<0.001 *
0–9	238 (0.6)	471 (1.1)	533 (1.2)	
10–19	996 (2.6)	1207 (2.8)	2107 (4.7)	
20–29	10,810 (28.0)	11,946 (27.7)	9560 (21.2)	
30–39	3431 (8.9)	5907 (13.7)	3580 (7.9)	
40–49	4792 (12.4)	5338 (12.4)	6164 (13.7)	
50–59	7958 (20.6)	8366 (19.4)	8476 (18.8)	
60–69	5977 (15.5)	6167 (14.3)	6732 (14.9)	
70–79	2469 (6.4)	2375 (5.5)	4865 (10.8)	
80+	1900 (4.9)	1412 (3.3)	3080 (6.8)	
Sex				<0.001 *
Male	13,716 (35.6)	17,847 (41.3)	19,216 (42.6)	
Female	24,855 (64.4)	25,342 (58.7)	25,881 (57.4)	
CCI score				<0.001 *
0	34,603 (89.7)	39,946 (92.5)	40,654 (90.2)	
1	2028 (5.3)	1743 (4.0)	2314 (5.1)	
≥2	1940 (5.0)	1500 (3.5)	2129 (4.7)	
Hypertension	7888 (20.5)	7552 (17.5)	10,257 (22.7)	<0.001 *
COVID-19	2836 (7.4)	2489 (5.8)	2618 (5.8)	<0.001 *
Prognosis of COVID-19				
Morbidity	185 (6.5)	161 (6.5)	212 (8.1)	0.032 *
Mortality	86 (0.23)	62 (0.14)	85 (0.19)	0.029 *

Abbreviations: CCI, Charlson comorbidity index; COVID-19, coronavirus disease 2019. * Chi-squared test. Significance at *p* < 0.05.

**Table 2:** Crude and adjusted odds ratios of the association of income with COVID-19 infection in the total participants.

Characteristics	COVID-19	Control	ORs (95% Confidence Interval) for COVID-19			
	(Exposure/Total, %)	(Exposure/Total, %)	Crude	*p*-Value	Adjusted ^†^	*p*-Value
Income group						
Low	2836/7943 (35.7%)	35,735/118,914 (30.1%)	1		1	
Middle	2489/7943 (31.3%)	40,700/118,914 (34.2%)	0.77 (0.73–0.82)	<0.001 *	0.78 (0.74–0.83)	<0.001 *
High	2618/7943 (33.0%)	42,479/118,914 (35.7%)	0.78 (0.74–0.82)	<0.001 *	0.79 (0.75–0.83)	<0.001 *
Income level (mean, SD)	10.00 (6.76)	10.75 (6.39)	0.98 (0.98–0.99)	<0.001 *	0.98 (0.98–0.99)	<0.001 *

* Logistic regression model, significance at *p* < 0.05. ^†^ Adjusted model for age, sex, CCI score and hypertension.

**Table 3:** Crude and adjusted odds ratios of the association of income with morbidity in COVID-19 participants.

Characteristics	Severe Participants	Mild Participants	ORs (95% Confidence Interval) for Morbidity			
	(Exposure/Total, %)	(Exposure/Total, %)	Crude	*p*-Value	Adjusted ^†^	*p*-Value
Income group						
Low	185/558 (33.2%)	2651/7385 (35.9%)	1		1	
Middle	161/558 (28.9%)	2328/7385 (31.5%)	0.99 (0.80–1.23)	0.936	1.21 (0.96–1.53)	0.108
High	212/558 (38.0%)	2406/7385 (32.6%)	1.26 (1.03–1.55)	0.026 *	1.17 (0.94–1.46)	0.172
Income level (mean, SD)	10.64 (7.21)	9.95 (6.73)	1.02 (1.00–1.03)	0.020 *	1.01 (1.00–1.03)	0.056

* Logistic regression model, significance at *p* < 0.05. ^†^ Adjusted model for age, sex, CCI score and hypertension.

**Table 4:** Crude and adjusted odds ratios of the association of income with mortality in COVID-19 participants.

Characteristics	Dead Participants	Survived Participants	ORs (95% Confidence Interval) for Mortality			
	(Exposure/Total, %)	(Exposure/Total, %)	Crude	*p*-Value	Adjusted ^†^	*p*-Value
Income group						
Low	86/233 (36.9%)	2750/7710 (35.7%)	1		1	
Middle	62/233 (26.6%)	2427/7710 (31.5%)	0.82 (0.59–1.14)	0.231	1.09 (0.75–1.58)	0.654
High	85/233 (36.5%)	2533/7710 (32.9%)	1.07 (0.79–1.46)	0.650	0.76 (0.54–1.08)	0.123
Income level (mean, SD)	10.07 (7.57)	10.00 (6.74)	1.00 (0.98–1.02)	0.876	0.99 (0.97–1.01)	0.148

^†^ Adjusted model for age, sex, CCI score and hypertension.

## Data Availability

Releasing the data by the researcher is not legally permitted. All data are available from the Korea Centers for Disease Control and Prevention database. The Korea Centers for Disease Control and Prevention database allows data access, at a particular cost, for any researcher who promises to follow the research ethic guidelines. The data of this article can be downloaded from the website after agreeing to follow the research ethic guidelines.

## Supplementary Materials

The following are available online at https://www.mdpi.com/article/10.3390/jcm10204733/s1, Supplementary Table S1: Distribution of national health insurance contributions in South Korea by income quintile. Supplementary Table S2: Subgroup analyses of crude and adjusted odds ratios of the association of income with COVID-19 infection in total participants by covariates. Supplementary Table S3: Subgroup analyses of crude and adjusted odds ratios of the association of income with morbidity in COVID-19 participants by covariates. Supplementary Table S4: Subgroup analyses of crude and adjusted odds ratios of the association of income with mortality in COVID-19 participants by covariates.
