# Supplementary material for: The Effects of Income Level on Susceptibility to COVID-19 and COVID-19 Morbidity/Mortality: A Nationwide Cohort Study in South Korea
Source: J Clin Med. 2021 Oct 15;10(20):4733. doi: 10.3390/jcm10204733 (PMC8541024; doi:10.3390/jcm10204733)
Supplement: Supplementary file 1 [file jcm-10-04733-s001.zip › jcm-1375646-supplementary.pdf]

Supplement Table S1. Distribution of national health insurance contributions in South Korea by income quintile

Income level	Households	Maximum contribution (KRW†)	Average contribution (KRW†)	Population under
Total	25,709,767	3,182,760	108,733	49,913,552
1	1,285,487	32,300	18,099	1,852,164
2	1,285,489	45,220	29,542	1,966,774
3	1,285,488	52,090	37,278	2,015,535
4	1,285,489	56,520	41,948	1,935,542
5	1,285,488	58,140	43,419	1,955,845
6	1,285,488	62,300	46,494	1,980,967
7	1,285,488	65,890	51,071	2,011,952
8	1,285,489	71,060	56,022	2,045,405
9	1,285,488	77,520	63,415	2,141,750
10	1,285,489	83,780	71,675	2,218,623
11	1,285,488	91,240	80,890	2,303,721
12	1,285,488	99,450	91,195	2,416,710
13	1,285,489	109,960	102,900	2,476,432
14	1,285,488	122,700	113,121	2,541,918
15	1,285,489	137,400	127,818	2,838,739
16	1,285,487	157,150	146,221	3,039,599
17	1,285,489	187,230	169,298	3,222,956
18	1,285,488	226,880	199,907	3,508,001
19	1,285,489	291,940	244,582	3,678,441
20	1,285,489	3,182,760	439,769	3,762,478

†KRW: South Korean Won

Supplement Table S2. Subgroup analyses of crude and adjusted odd ratios of income for COVID-19 infection in total participants by covariates

Characteristics	COVID-19	Control	ORs (95% confidence interval) for COVID-19			
	(exposure/total, %)	(exposure/total, %)	Crude	P-value	Adjusted †	*P*-value
**Age < 50 years old (n = 67,080)**						
Income group						
Low	1,416/4,200 (33.7%)	18,851/62,880 (30.0%)	1		1	
Middle	1,413/4,200 (33.6%)	23,456/62,880 (37.3%)	0.80 (0.74-0.87)	<0.001*	0.81 (0.75-0.87)	< .001*
High	1,371/4,200 (32.6%)	20,573/62,880 (32.7%)	0.89 (0.82-0.96)	0.002	0.89 (0.83-0.96)	.003*
Income level (mean, SD)	10.24 (6.52)	10.57 (6.13)	0.99 (0.99-1.00)	0.001	0.99 (0.99-1.00)	.002*
**Age ≥ 50 years old (n = 59,777)**						
Income group						
Low	1,420/3,743 (37.9%)	16,884/56,034 (30.1%)	1		1	
Middle	1,076/3,743 (28.7%)	17,244/56,034 (30.8%)	0.74 (0.68-0.81)	<0.001*	0.76 (0.70-0.83)	< .001*
High	1,247/3,743 (33.3%)	21,906/56,034 (39.1%)	0.68 (0.63-0.73)	<0.001*	0.69 (0.64-0.75)	< .001*
Income level	9.73 (7.01)	10.95 (6.66)	0.97 (0.97-0.98)	<0.001*	0.98 (0.97-0.98)	< .001*

(mean, SD)						
**Men (n = 50,779)**						
Income group						
Low	1,011/3,177 (31.8%)	12,705/47,602 (26.7%)	1		1	
Middle	997/3,177 (31.4%)	16,850/47,602 (35.4%)	0.74 (0.68-0.81)	<0.001*	0.77 (0.70-0.84)	< .001*
High	1,169/3,177 (36.8%)	18,047/47,602 (37.9%)	0.81 (0.75-0.89)	<0.001*	0.84 (0.77-0.91)	< .001*
Income level (mean, SD)	10.58 (6.82)	11.19 (6.30)	0.99 (0.98-0.99)	<0.001*	0.99 (0.98-0.99)	< .001*
**Women (n = 76,078)**						
Income group						
Low	1,825/4,766 (38.3%)	23,030/71,312 (32.3%)	1		1	
Middle	1,492/4,766 (31.3%)	23,850/71,312 (33.4%)	0.79 (0.74-0.85)	<0.001*	0.80 (0.74-0.86)	< .001*
High	1,449/4,766 (30.4%)	24,432/71,312 (34.3%)	0.75 (0.70-0.80)	<0.001*	0.75 (0.70-0.81)	< .001*
Income level (mean, SD)	9.61 (6.70)	10.46 (6.43)	0.98 (0.98-0.98)	<0.001*	0.98 (0.98-0.99)	< .001*
**CCI scores = 0 (n = 115,203)**						

Income group						
Low	2,217/6,413 (34.6%)	32,386/108,790 (29.8%)	1		1	
Middle	2,099/6,413 (32.7%)	37,847/108,790 (34.8%)	0.81 (0.76-0.86)	<0.001*	0.81 (0.76-0.86)	< .001*
High	2,097/6,413 (32.7%)	38,557/108,790 (35.4%)	0.79 (0.75-0.85)	<0.001*	0.81 (0.76-0.86)	< .001*
Income level (mean, SD)	10.15 (6.61)	10.77 (6.33)	0.99 (0.98-0.99)	<0.001*	0.99 (0.98-0.99)	< .001*
**CCI scores = 1 (n = 6,085)**						
Income group						
Low	340/875 (38.9%)	1,688/5,210 (32.4%)	1		1	
Middle	227/875 (25.9%)	1,516/5,210 (29.1%)	0.74 (0.62-0.89)	0.001*	0.72 (0.60-0.87)	.001*
High	308/875 (35.2%)	2,006/5,210 (38.5%)	0.76 (0.65-0.90)	0.002*	0.78 (0.66-0.93)	.005*
Income level (mean, SD)	9.64 (7.26)	10.60 (6.91)	0.98 (0.97-0.99)	<0.001*	0.98 (0.97-0.99)	.001*
**CCI scores ≥ 2 (n = 5,569)**						
Income group						
Low	279/655 (42.6%)	1,661/4,914 (33.8%)	1		1	

Middle	163/655 (24.9%)	1,337/4,914 (27.2%)	0.73 (0.59-0.89)	0.002*	0.74 (0.60-0.91)	.005*
High	213/655 (32.5%)	1,916/4,914 (39.0%)	0.66 (0.55-0.80)	<0.001*	0.65 (0.54-0.79)	< .001*
Income level (mean, SD)	9.02 (7.43)	10.54 (7.08)	0.97 (0.96-0.98)	<0.001*	0.97 (0.96-0.98)	< .001*
**Non-hypertension (n = 101,160)**						
Income group						
Low	2,206/6,247 (35.3%)	28,477/94,913 (30.0%)	1		1	
Middle	1,997/6,247 (32.0%)	33,640/94,913 (35.4%)	0.77 (0.72-0.82)	<0.001*	0.77 (0.73-0.82)	< .001*
High	2,044/6,247 (32.7%)	32,796/94,913 (34.6%)	0.80 (0.76-0.86)	<0.001*	0.81 (0.76-0.87)	< .001*
Income level (mean, SD)	10.04 (6.67)	10.69 (6.28)	0.98 (0.98-0.99)	<0.001*	0.99 (0.98-0.99)	< .001*
**Hypertension (n = 25,697)**						
Income group						
Low	630/1,696 (37.1%)	7,258/24,001 (30.2%)	1		1	
Middle	492/1,696 (29.0%)	7,060/24,001 (29.4%)	0.80 (0.71-0.91)	<0.001*	0.84 (0.74-0.95)	.005*
High	574/1,696 (33.8%)	9,683/24,001 (40.3%)	0.68 (0.61-0.77)	<0.001*	0.71 (0.63-0.80)	< .001*

Income level (mean, SD)	9.87 (7.11)	11.00 (6.79)	0.98 (0.97-0.98)	<0.001*	0.98 (0.97-0.99)	< .001*

* Logistic regression model, Significance at *P* < .05

† Adjusted model for age, sex, CCI scores and hypertension.

Supplement Table S3. Subgroup analyses of crude and adjusted odd ratios of income for morbidity in COVID-19 participants by covariates

Characteristics	Severe participants (exposure/total, %)	Mild participants (exposure/total, %)	ORs (95% confidence interval) for morbidity			
			Crude	P-value	Adjusted †	*P*-value
**Age < 50 years old (n = 4,200)**						
Income group						
Low	25/93 (26.9%)	1,391/4,107 (33.9%)	1		1	
Middle	37/93 (39.8%)	1,376/4,107 (33.5%)	1.50 (0.90-2.50)	0.124	1.59 (0.95-2.68)	.08
High	31/93 (33.3%)	1,340/4,107 (32.6%)	1.29 (0.76-2.19)	0.353	1.39 (0.81-2.38)	.24
Income level (mean, SD)	10.99 (6.25)	10.23 (6.53)	1.02 (0.99-1.05)	0.266	1.02 (0.99-1.06)	.15
**Age ≥ 50 years old (n = 3,743)**						
Income group						
Low	160/465 (34.4%)	1,260/3,278 (38.4%)	1		1	
Middle	124/465 (26.7%)	952/3,278 (29.0%)	1.03 (0.80-1.32)	0.842	1.13 (0.87-1.48)	.36
High	181/465 (38.9%)	1,066/3,278 (32.5%)	1.34 (1.07-1.68)	0.012*	1.06 (0.83-1.36)	.65
Income level	10.57 (7.39)	9.61 (6.95)	1.02 (1.01-1.03)	0.006*	1.01 (0.99-1.02)	.37

(mean, SD)						
**Men (n = 3,177)**						
Income group						
Low	90/299 (30.1%)	921/2,878 (32.0%)	1		1	
Middle	92/299 (30.8%)	905/2,878 (31.4%)	1.04 (0.77-1.41)	0.799	1.49 (1.06-2.07)	.02*
High	117/299 (39.1%)	1,052/2,878 (36.6%)	1.14 (0.85-1.52)	0.380	1.18 (0.86-1.62)	.31
Income level (mean, SD)	10.96 (7.05)	10.55 (6.80)	1.01 (0.99-1.03)	0.322	1.01 (0.99-1.03)	.19
**Women (n = 4,766)**						
Income group						
Low	95/259 (36.7%)	1,730/4,507 (38.4%)	1		1	
Middle	69/259 (26.6%)	1,423/4,507 (31.6%)	0.88 (0.64-1.21)	0.443	0.98 (0.71-1.37)	.93
High	95/259 (36.7%)	1,354/4,507 (30.0%)	1.28 (0.95-1.71)	0.101	1.15 (0.84-1.57)	.38
Income level (mean, SD)	10.28 (7.38)	9.57 (6.65)	1.02 (1.00-1.04)	0.098	1.01 (0.99-1.03)	.21
**CCI scores = 0 (n = 6,413)**						

Income group						
Low	70/257 (27.2%)	2,147/6,156 (34.9%)	1		1	
Middle	87/257 (33.9%)	2,012/6,156 (32.7%)	1.33 (0.96-1.83)	0.085	1.39 (1.01-1.93)	.05*
High	100/257 (38.9%)	1,997/6,156 (32.4%)	1.54 (1.13-2.10)	0.007*	1.47 (1.07-2.03)	.02*
Income level (mean, SD)	11.46 (6.68)	10.10 (6.60)	1.03 (1.01-1.05)	0.001*	1.03 (1.01-1.05)	.004*
**CCI scores = 1 (n = 875)**						
Income group						
Low	53/132 (40.2%)	287/743 (38.6%)	1		1	
Middle	30/132 (22.7%)	197/743 (26.5%)	0.83 (0.51-1.34)	0.434	0.91 (0.54-1.51)	.71
High	49/132 (37.1%)	259/743 (34.9%)	1.02 (0.67-1.56)	0.911	0.80 (0.51-1.26)	.34
Income level (mean, SD)	9.94 (7.72)	9.59 (7.18)	1.01 (0.98-1.03)	0.610	0.99 (0.97-1.02)	.57
**CCI scores ≥ 2 (n = 655)**						
Income group						
Low	62/169 (36.7%)	217/486 (44.7%)	1		1	

Middle	44/169 (26.0%)	119/486 (24.5%)	1.29 (0.83-2.02)	0.258	1.35 (0.84-2.18)	.22
High	63/169 (37.3%)	150/486 (30.9%)	1.47 (0.98-2.21)	0.064	1.04 (0.67-1.61)	.88
Income level (mean, SD)	9.95 (7.47)	8.69 (7.39)	1.02 (1.00-1.05)	0.058	1.00 (0.98-1.03)	.80
**Non-hypertension (n = 6,247)**						
Income group						
Low	87/280 (31.1%)	2,119/5,967 (35.5%)	1		1	
Middle	93/280 (33.2%)	1,904/5,967 (31.9%)	1.19 (0.88-1.60)	0.255	1.41 (1.03-1.93)	.03*
High	100/280 (35.7%)	1,944/5,967 (32.6%)	1.25 (0.93-1.68)	0.133	1.28 (0.94-1.74)	.12
Income level (mean, SD)	10.75 (6.94)	10.00 (6.65)	1.02 (1.00-1.04)	0.066	1.02 (1.00-1.04)	.03*
**Hypertension (n = 1,696)**						
Income group						
Low	98/278 (35.3%)	532/1,418 (37.5%)	1		1	
Middle	68/278 (24.5%)	424/1,418 (29.9%)	0.87 (0.62-1.22)	0.417	0.99 (0.69-1.41)	.94
High	112/278 (40.3%)	462/1,418 (32.6%)	1.32 (0.98-1.77)	0.071	1.03 (0.75-1.42)	.87

Income level (mean, SD)	10.53 (7.48)	9.74 (7.02)	1.02 (1.00-1.04)	0.090	1.00 (0.98-1.02)	.78

* Logistic regression model, Significance at $P < .05$

† Adjusted model for age, sex, CCI scores and hypertension.

Supplement Table S4. Subgroup analyses of crude and adjusted odd ratios of income for mortality in COVID-19 participants by covariates

Characteristics	Dead participants (exposure/total, %)	Survived participants (exposure/total, %)	ORs (95% confidence interval) for mortality			
			Crude	P-value	Adjusted †	*P*-value
**Age < 50 years old (n = 4,200)**						
Income group						
Low	1/3 (33.3%)	1,415/4,197 (33.7%)	1		1	
Middle	1/3 (33.3%)	1,412/4,197 (33.6%)	1.00 (0.06-16.04)	0.999	1.21 (0.07-20.37)	.90
High	1/3 (33.3%)	1,370/4,197 (32.6%)	1.03 (0.07-16.53)	0.982	1.20 (0.07-21.16)	.90
Income level (mean, SD)	11.00 (9.17)	10.24 (6.52)	1.02 (0.86-1.21)	0.841	1.03 (0.87-1.24)	.71
**Age ≥ 50 years old (n = 3,743)**						
Income group						
Low	85/230 (37.0%)	1,335/3,513 (38.0%)	1		1	
Middle	61/230 (26.5%)	1,015/3,513 (28.9%)	0.94 (0.67-1.33)	0.739	1.09 (0.75-1.58)	.66
High	84/230 (36.5%)	1,163/3,513 (33.1%)	1.13 (0.83-1.55)	0.428	0.76 (0.54-1.08)	.12
Income level	10.06 (7.57)	9.71 (6.98)	1.01 (0.99-1.03)	0.464	0.99 (0.97-1.01)	.14

(mean, SD)						
**Men (n = 3,177)**						
Income group						
Low	43/131 (32.8%)	968/3,046 (31.8%)	1		1	
Middle	40/131 (30.5%)	957/3,046 (31.4%)	0.94 (0.61-1.46)	0.786	1.49 (0.91-2.46)	.12
High	48/131 (36.6%)	1,121/3,046 (36.8%)	0.96 (0.63-1.47)	0.864	0.78 (0.48-1.26)	.31
Income level (mean, SD)	10.37 (7.36)	10.59 (6.80)	1.00 (0.97-1.02)	0.709	0.99 (0.96-1.01)	.26
**Women (n = 4,766)**						
Income group						
Low	43/102 (42.2%)	1,782/4,664 (38.2%)	1		1	
Middle	22/102 (21.6%)	1,470/4,664 (31.5%)	0.62 (0.37-1.04)	0.071	0.72 (0.41-1.27)	.26
High	37/102 (36.3%)	1,412/4,664 (30.3%)	1.09 (0.70-1.70)	0.717	0.75 (0.46-1.24)	.27
Income level (mean, SD)	9.69 (7.85)	9.61 (6.67)	1.00 (0.97-1.03)	0.910	0.99 (0.96-1.02)	.38
**CCI scores = 0 (n = 6,413)**						

Income group						
Low	16/63 (25.4%)	2,201/6,350 (34.7%)	1		1	
Middle	21/63 (33.3%)	2,078/6,350 (32.7%)	1.39 (0.72-2.67)	0.323	1.59 (0.79-3.20)	.19
High	26/63 (41.3%)	2,071/6,350 (32.6%)	1.73 (0.92-3.23)	0.087	1.31 (0.67-2.57)	.43
Income level (mean, SD)	11.84 (6.80)	10.13 (6.61)	1.04 (1.00-1.08)	0.043*	1.02 (0.98-1.06)	.33
**CCI scores = 1 (n = 875)**						
Income group						
Low	29/61 (47.5%)	311/814 (38.2%)	1		1	
Middle	13/61 (21.3%)	214/814 (26.3%)	0.65 (0.33-1.28)	0.215	0.69 (0.33-1.43)	.32
High	19/61 (31.1%)	289/814 (35.5%)	0.71 (0.39-1.29)	0.254	0.43 (0.22-0.83)	.01*
Income level (mean, SD)	8.89 (8.04)	9.70 (7.20)	0.99 (0.95-1.02)	0.399	0.96 (0.92-0.99)	.02*
**CCI scores ≥ 2 (n = 655)**						
Income group						
Low	41/109 (37.6%)	238/546 (43.6%)	1		1	

Middle	28/109 (25.7%)	135/546 (24.7%)	1.20 (0.71-2.04)	0.488	1.25 (0.71-2.21)	.44
High	40/109 (36.7%)	173/546 (31.7%)	1.34 (0.83-2.16)	0.227	0.85 (0.51-1.43)	.55
Income level (mean, SD)	9.71 (7.60)	8.88 (7.39)	1.02 (0.99-1.04)	0.288	0.99 (0.96-1.02)	.47
**Non-hypertension (n = 6,247)**						
Income group						
Low	28/74 (37.8%)	2,178/6,173 (35.3%)	1		1	
Middle	24/74 (32.4%)	1,973/6,173 (32.0%)	0.95 (0.55-1.64)	0.843	1.32 (0.71-2.45)	.37
High	22/74 (29.7%)	2,022/6,173 (32.8%)	0.85 (0.48-1.48)	0.560	0.57 (0.30-1.09)	.09
Income level (mean, SD)	9.62 (7.57)	10.04 (6.66)	0.99 (0.96-1.03)	0.591	0.98 (0.94-1.01)	.17
**Hypertension (n = 1,696)**						
Income group						
Low	58/159 (36.5%)	572/1,537 (37.2%)	1		1	
Middle	38/159 (23.9%)	454/1,537 (29.5%)	0.83 (0.54-1.27)	0.379	0.96 (0.60-1.52)	.85
High	63/159 (39.6%)	511/1,537 (33.2%)	1.22 (0.84-1.77)	0.308	0.86 (0.57-1.29)	.46

Income level (mean, SD)	10.28 (7.58)	9.62 (7.57)	1.01 (0.99-1.03)	0.450	0.99 (0.97-1.01)	.42

* Logistic regression model, Significance at *P* < .05

† Adjusted model for age, sex, CCI scores and hypertension.
